# Oxygen versus Reactive Oxygen in the Regulation of HIF-1**α**: The Balance Tips

**DOI:** 10.1155/2012/436981

**Published:** 2012-10-09

**Authors:** Thilo Hagen

**Affiliations:** Department of Biochemistry, National University of Singapore, 117596, Singapore

## Abstract

Hypoxia inducible factor (HIF) is known as the master regulator of the cellular response to hypoxia and is of pivotal importance during development as well as in human disease, particularly in cancer. It is composed of a constitutively expressed **β** subunit (HIF-1**β**) and an oxygen-regulated **α** subunit (HIF-1**α** and HIF-2**α**), whose stability is tightly controlled by a family of oxygen- and iron-dependent prolyl hydroxylase enzymes. Whether or not mitochondria-derived reactive oxygen species (ROS) are involved in the regulation of Hypoxia Inducible Factor-1**α** has been a matter of contention for the last 10 years, with equally compelling evidence in favor and against their contribution. A number of recent papers appear to tip the balance against a role for ROS. Thus, it has been demonstrated that HIF prolyl hydroxylases are unlikely to be physiological targets of ROS and that the increase in ROS that is associated with downregulation of Thioredoxin Reductase in hypoxia does not affect HIF-1**α** stabilization. Finally, the protein CHCHD4, which modulates cellular HIF-1**α** concentrations by promoting mitochondrial electron transport chain activity, has been proposed to exert its regulatory effect by affecting cellular oxygen availability. These reports are consistent with the hypothesis that mitochondria play a critical role in the regulation of HIF-1**α** by controlling intracellular oxygen concentrations.

## 1. Introduction

The response of cells to hypoxia involves one of the best understood cellular signaling pathways. Research over the last 15 years has shown that the primary response is mediated via Hypoxia Inducible Factor, HIF, a dimeric transcription factor that was discovered in 1992 by Semenza and Wang [1]. HIF is composed of two subunits, an oxygen inducible *α* and a constitutively expressed *β* subunit, HIF-1*α*, and HIF-1*β*, respectively. As frequently is the case for key mediators in various signaling pathways, HIF-1*α* is primarily regulated at the level of its protein stability. Thus, cells constitutively transcribe and translate HIF-1*α*. However, cellular HIF-1*α* is normally almost undetectable because of an extremely rapid rate of HIF-1*α* protein ubiquitination and subsequent proteasome-dependent degradation under normoxic conditions. Lack of oxygen leads to blocking of HIF-1*α* ubiquitination, resulting in rapid protein accumulation and activation of the HIF transcriptional response.

It was observed in the late 1990s that a functional electron transport chain is required for hypoxia-dependent HIF-1*α* stabilization, and this led to the suggestion that reactive oxygen species (ROS), released from the ETC, are involved in sensing of the cellular oxygen concentration [2, 3]. This would appear logical given the role of mitochondria as the major consumers of cellular oxygen and the well-known signaling function of ROS, in particular of H_2_O_2_. However, a series of classic papers at the beginning of the last decade [4–7] identified the hydroxylation of two proline residues, mediated by a family of oxygen-dependent prolyl-4-hydroxylase domain enzymes (PHDs), as the mechanism accounting for the regulation of the HIF-1*α* protein by oxygen. Upon oxygen-dependent hydroxylation of Pro402 or Pro564, HIF-1*α* was shown to bind to the pVHL-Elongin B/C-Cul2 E3 ubiquitin ligase, leading to its ubiquitination and subsequent degradation. Consequently, inhibition of PHD activity due to either lack of oxygen or due to various chemical inhibitors leads to HIF-1*α* accumulation.

Nevertheless, how exactly mitochondria contribute to this mechanism remained highly contentious and a series of papers in 2005 reported the requirement of ROS, produced by complex III of the mitochondrial electron transport chain, for hypoxia-dependent HIF-1*α* stabilization [8–10]. This conclusion was reached by using a number of genetic and pharmacological interventions to manipulate electron transport chain-dependent ROS production and was further supported by follow-up studies [11]. According to this so called “ROS" hypothesis, hypoxia causes the production of superoxide at respiratory complex III. The superoxide, likely upon its superoxide dismutase (SOD) dependent conversion to H_2_O_2_, has been proposed to function to directly inhibit PHD enzymes by oxidizing the essential nonheme-bound iron.

In an alternative mechanism, the activity of the mitochondrial ETC was proposed to function by regulating the cellular oxygen availability [12–15]. Mitochondria are the major cellular sink for oxygen. According to the “oxygen” hypothesis, a decrease in the rate of the electron transport chain activity of mitochondria results in an increase in the cytoplasmic oxygen concentration. This in turn leads to PHD reactivation and destabilization of HIF-1*α*. Notably, the HIF-1*α* homolog HIF-2*α* has been reported to be also regulated via this mechanism [16]. Thus, the authors reported that inhibiting mitochondrial function regulates HIF-2*α* via changes in mitochondrial oxygen consumption but not mitochondrial ROS production.

**Figure 1:**
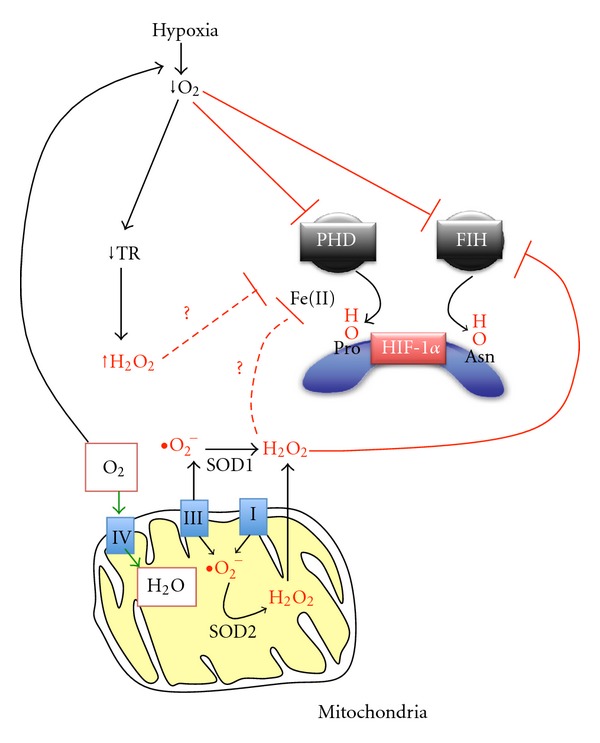
Mitochondria-dependent control of intracellular oxygen and ROS levels and its role in the regulation of HIF-1*α*. Mitochondria, via complex IV of the electron transport chain, are the major consumers of cellular oxygen. Under conditions of limiting oxygen diffusion, mitochondrial respiratory activity therefore exerts control over the intracellular oxygen concentration. Consistently, gradients in the oxygen concentration between extracellular space, cytoplasm, and perimitochondrial space have been observed [34, 35]. Changes in the intracellular oxygen concentration are sensed by oxygen-dependent dioxygenase enzymes, prolyl hydroxylase domain enzymes (PHD), and the asparagine hydroxylase Factor Inhibiting HIF (FIH). The major target of these two oxygen sensing enzyme classes is the transcription factor Hypoxia Inducible Factor-1*α* (HIF-1*α*). PHD enzymes and FIH hydroxylate HIF-1*α* at specific proline and asparagine residues to induce HIF-1*α* protein ubiquitination and degradation and inhibit its transcriptional activity, respectively. Under low oxygen conditions, PHD and FIH are inhibited, hence leading to activation of the hypoxic response. In most cell types in addition to consuming oxygen, the mitochondrial electron transport chain is also the major producer of superoxide which is converted into membrane permeable and diffusible H_2_O_2_ by Superoxide Dismutase 1 and 2 (SOD1 and SOD2). It has been proposed that mitochondrial production of ROS derived from respiratory complex III is increased under hypoxia, and these ROS contribute to HIF-1*α* protein stabilization by inhibiting PHD enzymes. However, recent studies indicate that PHD enzymes have very low sensitivity to H_2_O_2_ while FIH is much more susceptible to inactivation by peroxide [18]. Furthermore, it has been shown that hypoxia leads to downregulation of thioredoxin reductase 1 (TR1) and consequently to increased intracellular H_2_O_2_ concentrations [17]. However, manipulation of TR1 expression in hypoxia was without effects on HIF-1*α* accumulation and activation. These results provide further support that PHD activity towards HIF-1*α* in hypoxia is primarily controlled by intracellular oxygen concentrations.

Because most pharmacological and genetic interventions to alter the function of the electron transport chain induce changes in both oxygen consumption and mitochondrial ROS production, it has been difficult to provide conclusive evidence for the validity of either hypothesis. Notably, a recent report in PLoS One has taken an interesting alternative approach [17]. In their study, the authors determine how changes in the activity of the thioredoxin/thioredoxin reductase system, one of the two major cellular scavenging systems of H_2_O_2_, affect hypoxia-dependent HIF-1*α* stabilization. Interestingly, the study found that Thioredoxin Reductase 1 (TR1) is downregulated at the mRNA and protein level under conditions of hypoxia in two different cell types (EMT6 breast cancer cells and DT6 transformed fibroblasts). The authors show that HIF is not involved in hypoxia-dependent TR1 downregulation. Thus, activation of HIF by treatment of cells with PHD inhibitors is not sufficient to reduce TR1 expression and blocking HIF activation in hypoxia by siRNA-mediated silencing of HIF-1*α* does not prevent TR1 downregulation in hypoxia. The hypoxia-dependent TR1 downregulation was shown to be important for maintaining high levels of ROS under hypoxic conditions. Thus, TR1 knockdown cells were found to show a larger accumulation of H_2_O_2_ in hypoxia while TR1 overexpression blocked hypoxic generation of ROS. However, importantly, these interventions were without effect on hypoxia-dependent HIF-1*α* stabilization in both studied cell types. Silencing or overexpression of TR1 was also without effect on HIF transcriptional activity, as determined by measuring the mRNA levels of the HIF target genes VEGF and adrenomedullin. These results would therefore suggest that hypoxic generation of ROS is not required for the hypoxia response (Figure 1).

According to the ROS hypothesis, H_2_O_2_, generated under hypoxia, would inhibit the activity of prolyl hydroxylase enzymes, possibly by oxidation of the nonheme Fe(II) that is essential for PHD function. A recent study in EMBO Reports has looked at this potential mechanism in detail [18]. The authors show that perhaps contrary to expectations, HIF prolyl hydroxylases have low sensitivity to inhibition by H_2_O_2_. Interestingly, Factor Inhibiting HIF (FIH), a 2-oxoglutarate-dependent dioxygenase which belongs to the same family as PHD enzymes, is much more susceptible to H_2_O_2_-dependent inactivation. In the presence of oxygen, FIH hydroxylates an asparagine residue in the carboxy-terminal transactivation domain of HIF-1*α* and its homolog, HIF-2*α*. This posttranslational modification leads to inhibition of HIF transcriptional activity in normoxia, an effect that is reversed at low oxygen concentrations. In their study, the authors treated various cell types for short periods of time with low micromolar concentrations of peroxides (tert-butylhydroperoxide or H_2_O_2_). They then assayed PHD and FIH activity using antibodies which recognize hydroxylated proline or asparagine residues in HIF-1*α* or using mass spectrometry. Peroxides caused a dramatic inhibition of FIH dependent asparagine hydroxylation of HIF-1*α* as well as of a further FIH substrate, rabankyrin 5. In contrast, peroxide treatment resulted in only minor decreases in HIF-1*α* prolyl hydroxylation. Peroxide dependent FIH inhibition is most likely a consequence of a direct modification of FIH, as FIH remained inactive after immunoprecipitation from lysates of peroxide-treated cells. However, by using iron chelators, the authors show that FIH modification is not directly due to H_2_O_2_ but likely mediated through H_2_O_2_ undergoing iron dependent Fenton chemistry (Figure 1).

These results put a big question mark on the role of ROS generated under hypoxia to inhibit PHD activity. The dramatic inhibition of HIF-1*α* prolyl hydroxylation in hypoxia does not correlate with the modest effects of peroxide on prolyl hydroxylation. What is more, there is no consensus as to whether hypoxia actually causes an increase or a decrease in mitochondrial ROS production [19–21]. For instance, Waypa et al. [22] and Bell et al. [23] used a redox-sensitive, ratiometric green fluorescent protein sensor (RoGFP) which contains engineered cysteine residues that enable dithiol formation in response to oxidant stress. They found that hypoxia increases the RoGFP fluorescence in the cytoplasm and the mitochondrial intermembrane space. On the other hand, a study by Wang et al. [24] developed a mitochondria-targeted, circularly permuted yellow fluorescent protein-based superoxide sensor. Using this tool, they observed decreased superoxide flash frequency in anoxia or mild hypoxia, conditions where HIF-1*α* is stabilized. In contrast, reoxygenation resulted in a rapid increase in superoxide back to normal levels under conditions where HIF-1*α* is rapidly degraded. Hence, there is no correlation between mitochondrial superoxide production and HIF-1*α* stability. One caveat is that the fluorescent superoxide sensor detects only superoxide that is released into the matrix but not that released into the intermembrane space [25]. Interestingly, it has been observed that neurons lacking the mitochondrial isoform of superoxide dismutase (Mn-SOD), which have increased levels of matrix superoxide, can only survive culture in hypoxia, but not in normoxia [26]. One plausible explanation for this finding would be that in hypoxia, there is less superoxide production by the mitochondria. However, alternative explanations are possible [27]. Finally, a plethora of studies has shown that inhibition of the mitochondrial F_0_F_1_-ATPase leads to an increase in the mitochondrial membrane potential and slowing down of electron transport. This in turn results in increased half life of reduced, reactive intermediates in the electron transport chain, and consequently an increase in superoxide production from all superoxide producing sites within the electron transport chain. However, despite increased mitochondrial ROS production, F_0_F_1_-ATPase inhibition prevents HIF-1*α* stabilization in hypoxia [28, 29].

As evident from these examples, in order to distinguish between the mitochondria dependent regulation of HIF-1*α* proline hydroxylation via oxygen versus ROS, it would be desirable to manipulate the two parameters independently. This has thus far proved difficult to achieve. As a case in point, a recent study by Yang et al. [30], published in the Journal of Clinical Investigation, identified a new regulator of mitochondrial oxygen consumption, the protein CHCHD4. CHCHD4, which stands for Coiled-Coil-Helix-Coiled-Coil-Helix Domain Containing 4, exists as two transcript variants. The two variants encode for proteins that only differ in their amino terminus. Isoform 1 is also known as MIA40. It forms part of the mitochondrial disulfide relay system important for the import and oxidative folding of proteins in the mitochondrial intermembrane space [31]. Yang et al. found that CHCHD4 is critically involved in the regulation of HIF-1*α*. Thus, reducing CHCHD4 protein expression inhibited HIF-1*α* protein expression in hypoxia, while increased expression led to greater HIF-1*α* induction. These effects were a consequence of altered HIF-1*α* protein stability. Importantly, changes in HIF-1*α* protein stability correlated with effects on cellular oxygen consumption. Thus, overexpression of CHDHD4 led to increased mitochondrial respiratory rates which is expected to result in decreased intracellular oxygen concentrations, thus potentially explaining the observed greater HIF-1*α* stabilization in hypoxia. On the contrary, knockdown of both CHDHD4 isoforms inhibits oxygen consumption in the mitochondria, leading to increased intracellular oxygen availability. This again correlates with the observed inhibition of HIF-1*α* protein expression in hypoxia. The authors indeed suggest that regulation of HIF-1*α* by CHCHD4 is mediated by changes in the intracellular oxygen availability and not a consequence of altered levels of complex III derived ROS. This is based on their finding that the enhanced stabilization of HIF-1*α* protein in hypoxia upon CHCHD4 overexpression was not sensitive to the antioxidant N-acetylcysteine. However, to further confirm this conclusion, it would be important to determine the effect of CHCHD4 overexpression or silencing on cellular ROS concentrations, particularly in hypoxia. Another useful experiment would be to use mitochondria-targeted antioxidants (e.g., MitoQ, [32]) instead of the general antioxidant N-acetylcysteine, although these mitochondria-directed agents have also been reported to affect oxygen consumption rates [33].

Similar to previously published approaches, the manipulations in CHCHD4 expression in the experiments by Yang et al. may affect not only oxygen consumption rates, but also mitochondria derived ROS and are thus unable to conclusively distinguish between the two mechanisms. There are two notable exceptions where effects on mitochondrial respiration and superoxide production were uncoupled. In a study by Bell et al. [11], the authors used cells lacking cytochrome b, a critical electron transporting subunit in complex III. These cells are respiratory incompetent, but display a similar increase in intracellular H_2_O_2_ levels in hypoxia versus normoxia compared to wild type cells. According to the “oxygen” hypothesis, the increased intracellular oxygen availability in the cells should inhibit HIF-1*α* accumulation in hypoxia. However, on the contrary, HIF-1*α* protein accumulated similarly in wild type and cytochrome b deficient cells, suggesting that ROS play a role in HIF-1*α* stabilization in hypoxia. In contrast, in a recent study, we utilized alternative oxidase from *Ciona intestinalis* which upon overexpression transfers electrons directly from coenzyme Q to oxygen to form water, thus bypassing mitochondrial complex III [15]. As a result, superoxide production from complex III decreases while oxygen consumption is maintained. We found no difference when measuring HIF-1*α* stabilization in hypoxia in alternative oxidase overexpressing cells compared to control cells, thus arriving at the opposite conclusion that complex III derived ROS are not involved in HIF-1*α* stabilization upon exposure of cells to hypoxia.

Thus, although there still remains conflicting evidence, the described recent studies provide complementary evidence in favor of the hypothesis that mitochondrial oxygen consumption, but not mitochondria derived ROS, plays a major role in regulating HIF-1*α* protein levels in hypoxia. When taken together, the various studies confirm that prolyl hydroxylases, with their Km for oxygen in the range of the atmospheric oxygen concentration, are physiologically predominantly regulated by cellular oxygen availability. The design of this signaling cascade is clearly one of the most direct and most impressive examples of how cells can respond to signals in a highly specific and rapid manner.
